# Mobilizing health equity through Computable Biomedical Knowledge (CBK): a call to action to the library, information sciences, and health informatics communities

**DOI:** 10.5195/jmla.2024.1836

**Published:** 2024-04-01

**Authors:** Nancy J. Allee, Gerald Perry, Gabriel R. Rios, Joshua C. Rubin, Vignesh Subbian, Deborah E. Swain, Terrie R. Wheeler

**Affiliations:** 1 nallee@umich.edu, Director, Taubman Health Sciences Library & STEM, University of Michigan Library, Joint Faculty, Department of Learning Health Sciences, University of Michigan Medical School, University of Michigan, Ann Arbor, MI; 2 jerryperry@arizona.edu, Associate Dean Emeritus, University Libraries, University of Arizona, Tucson, AZ; 3 grrios@iu.edu, Library Director, Ruth Lilly Medical Library, Indiana University School of Medicine, Indianapolis, IN; 4 josh@joshcrubin.com, Program Officer for Learning Health System Initiatives, Department of Learning Health Sciences, University of Michigan Medical School, University of Michigan, Ann Arbor, MI; 5 vsubbian@arizona.edu, Associate Professor, College of Engineering, University of Arizona, Tucson, AZ; 6 dswain@nccu.edu, Professor, School of Library and Information Sciences, North Carolina Central University, Durham, NC; 7 tew2004@med.cornell.edu, Library Director, Samuel J. Wood Library, Weill Cornell Medicine, New York, NY

**Keywords:** Health Equity, Health Inequalities, Health Status Disparities, Computing Methodologies, Algorithms, Artificial Intelligence, Machine Learning, Library Science, Information Science

## Abstract

The twin pandemics of COVID-19 and structural racism brought into focus health disparities and disproportionate impacts of disease on communities of color. Health equity has subsequently emerged as a priority. Recognizing that the future of health care will be informed by advanced information technologies including artificial intelligence (AI), machine learning, and algorithmic applications, the authors argue that to advance towards states of improved health equity, health information professionals need to engage in and encourage the conduct of research at the intersections of health equity, health disparities, and computational biomedical knowledge (CBK) applications. Recommendations are provided with a means to engage in this mobilization effort.

The twin pandemics of COVID-19 and structural racism have surfaced and brought into sharp focus critical health disparities and disproportionate impacts of disease on communities of color [1, 2, 3, 4]. Health equity as an area of scholarship and activism has subsequently emerged as a priority [5, 6, 7, 8, 9]. Recognizing that the future of health care will be informed by advanced information technologies including artificial intelligence (AI), machine learning, and algorithmic applications, [10] the authors, all active in the Mobilizing Computable Biomedical Knowledge (MCBK) community, [11] argue that in order to advance towards states of improved health equity, knowledge workers, health and biomedical researchers, healthcare practitioners, government agencies, philanthropy, industry, consumer health advocacy and community-based organizations all need to be engaged in and encouraging the conduct of research at the intersections of health equity, health disparities, and computable biomedical knowledge (CBK) applications. CBK broadly encompasses knowledge related to human health that is explicit and machine interpretable. Examples include machine-readable and processable clinical care guidelines, predictive and interpretable models, calculators, statistical and logic models, among others [12, 13]. Accessible examples can be found through the PCBK, described as “a public repository mobilizing Computable Biomedical Knowledge artifacts,” [14] or the University of Michigan's Knowledge Grid [15].

CBK allows for knowledge to be “represented and reasoned upon using logic, formal standards, and mathematical approaches” [16]. In this perspective article, we call on health information professionals, including librarians and informaticians as specialized knowledge workers, to join and fully engage in this work and the surrounding movement to bring advanced computational and information technologies to bear on improving health care delivery, outcomes, and ultimately health equity. We believe the library and information science (LIS) communities have relevant, tangible skills to contribute. CBK as artifacts need to be curated and preserved, archived, deposited into accessible repositories, described using metadata, and rendered findable – all activities aligned with the skills and approaches commonly deployed by librarians and information science professionals.

## HEALTH EQUITY AND COMPUTABLE BIOMEDICAL KNOWLEDGE

Health equity and computable biomedical knowledge are interconnected, as access to accurate and comprehensive biomedical knowledge is critical to achieving health equity. CBK, which refers to knowledge representations that are both machine readable and actionable, can provide valuable insights about health disparities. That knowledge can inform models that clarify patterns and trends, potentially predicting outcomes based on factors such as race, ethnicity, socioeconomic status, or geographic location. In addition, CBK can also help to develop more timely and targeted interventions to address health disparities. For instance, by analyzing evidence on the effectiveness of different treatments for specific subpopulations, researchers can identify which interventions are most effective for addressing health disparities.

The authors argue that it is important to ensure that CBK is accessible to all communities and that it is used in a way that promotes health equity. This means that efforts must be made to address biases and ensure that data used to inform CBK artifacts are collected and analyzed in ways that are inclusive and representative of all communities. Additionally, efforts must be made to ensure that the insights gained from CBK are used to develop interventions that are accessible and appropriate for all communities, particularly those that have historically been marginalized or underserved.

Access to evidence is a critical component of health equity and is advanced by open frameworks in healthcare. Without access to accurate, reliable, and timely information, individuals and communities may face barriers to accessing healthcare services and to making informed decisions about their health and advocating for their own health needs.

## THE HEALTH EQUITY IMPERATIVE

As cited, a body of literature has emerged describing the disproportionate impacts of the COVID-19 pandemic on Black, Indigenous, and People of Color (BIPOC) [17], and a related corpus has emerged documenting pervasive BIPOC mistrust of healthcare writ large and healthcare delivery, specifically, including COVID-19 related treatment [18, 19]. At the same time, popular media [20, 21, 22] and scientific venues [23] have published and broadcasted findings that AI, machine learning, and algorithmic applications can and do perpetuate biases, including harmful racist tropes. Based on these knowledge sources, we see a need to act grounded in the values of both the medical and LIS professions, and an understanding of ethics of care as a frame of reference for advancing moral action.

Briefly, an ethics of care approach is centered around relationships and dependencies between individuals. It encourages us to consider the notions of “care” and “compassion” as moral behaviors [24]. Within the healthcare realm, to care about and have compassion for individuals potentially impacted by disease and illness become moral imperatives [25]. If we recognize the uncontested moral assertion that all individuals equally deserve care and compassion, then health equity must be fundamental to healthcare.

Within the LIS realm, to care and have compassion for the information and decision-making needs of individuals similarly becomes a moral imperative. For health information professionals these imperatives converge. Further, if we recognize that the future of healthcare will be manifestly informed by emerging advanced information technologies such as AI and ML, [26] then those technologies must be interrogated for the degrees to which they advance health equity. These are the imperatives that drive the need for LIS professionals to engage in and lead CBK-related work.

## LIBRARIANSHIP AND THE MCBK COMMUNITY

The MCBK community of practice was largely launched at a foundational meeting held in 2017 in Ann Arbor, MI, sponsored by the Department of Learning Health Systems at the University of Michigan (UM) [27]. The founding leaders were prescient in inviting thought leaders from the health sciences library community to participate in that meeting, recognizing that health sciences librarians' roles and expertise in organizing and providing access to evidence-based knowledge was foundational to the work of the movement. According to the MCBK Manifesto “Knowledge has the potential to improve healthcare, the health of individuals, and the health of populations. Every decision affecting health should be informed by the best available knowledge” [28].

As the MCBK movement was launched, those LIS thought leaders became active in the leadership of the effort, taking roles on the Steering Committee and co-chairing and serving as members of the various MCBK Working Groups that eventually emerged [29]. Those librarians quickly became advocates within the movement for explicit engagement in health equity issues as they related to CBK, honing in on the MCBK Manifesto's equity statement: “For moral and ethical reasons, it is imperative that each and every member of society have access to what is known at the time they are making health-related choices and decisions” [30].

## CALL TO ACTION

The authors advocate that research and engagement at the intersections of advancing health equity, reducing health disparities, and mobilizing CBK need to be equitably and transparently organized and structured to involve impacted stakeholders in ways that recognize and prioritize the interests of communities most adversely impacted by health disparities. We see this as fundamental to an ethics of care-informed approach [31] to solving for the persistent problem of equitable representation in the development of solutions to complex, often intractable problems in healthcare. The conduct of research at the intersections of health equity, health disparities, and CBK should address unequal economic and power dynamics and seek to establish a level healthcare playing field. Given the profound and widespread levels of mistrust around healthcare and biomedical research among communities of color, [32, 33, 34, 35, 36] failure to approach CBK with anything less than a humble, anti-racism strategy would be untenable, unethical and risk rendering the promises of CBK moot for entire communities.

The authors are addressing this call to action to LIS professionals because we see very clear connections between the fundamental work of health sciences librarianship, including the culture and values of the profession as articulated in codes of practice and ethics [37, 38], with the goals of the MCBK movement [39]. Those values include commitments to: diversity, equity, inclusion, and antiracism [40, 41], open science including open access, data, and research [42, 43], and longstanding commitments to dynamic emerging roles for librarians in the work areas of metadata, repositories (including knowledge bases), information stewardship and knowledge management, instruction, and engagement through outreach with impacted communities, especially those who have been consistently, structurally marginalized, minoritized, and oppressed. Librarians as boundary spanners [44] and champions for open access are well-positioned to help lead the MCBK community to consider the issues of health equity and equitable protocols for problem-solving engagement.

Both the LIS and MCBK communities have recognized the need to address equity in the provision of healthcare. The authors believe that the twin pandemics of COVID-19 and racism add urgency to the need for proactive engagement by the library and information sciences community in MCBK-related work. We believe librarians can especially contribute to “Mobilizing” functional work that is needed, and we believe that work must start as outreach to the LIS community. We see this as imperative and the ethical thing to do. We recognize the complexity of issues at hand and the need to advocate. We write with the purpose of encouraging the library, information sciences and health informatics communities to recognize the importance of engagement in this work and to act now.

These needs are clearly in evidence as we consider recent public health emergencies. We posit if there ever was a need for evidence to substantiate the potential for LIS involvement in health equity and CBK research and advocacy, the twin pandemics of COVID-19 and racism offer such and in consequence, we offer the following recommendations.

## RECOMMENDATIONS

1. LIS professionals must support, and amplify the need to support, health disparities research using ethics of care approaches. We must also expand our focus to consider the search for solutions, embracing the “Quintuple Aim” of transforming patient care to include improving patient experiences, seeking better outcomes, reducing costs, better ensuring clinician well-being, and embracing health equity[45]. One way to do this is by taking a holistic and inclusive view of CBK artifacts, i.e., considering the interdependencies between the technical and social components involved in the development and deployment of CBK artifacts. We believe MCBK is a solution in that advanced technological CBK artifacts, when created with an explicit consciousness of diversity, equity, inclusion, and antiracism values, can be used to deliver at scale bias-resistant benefits to the users and consumers of CBK.
2. LIS professionals must commit to training and educating the future research workforce in areas related to CBK and health equity. This can be done by applying domain skills in instruction and outreach to formal undergraduate and graduate training programs.
3. Health equity should not be an afterthought in biomedical research, particularly during the experimental design stage. Researchers engaged in CBK-based solutions need to better define what is health equity in their specific MCBK context. How is health equity advanced? This can be done by asking: whose experiences are centered in the data, application, algorithm, model, or artifact we are generating or using? Whose experiences are explicitly or implicitly missing?
4. LIS professionals, must ask, how do we involve the most significantly, and, potentially, severely impacted constituencies whose experiences are the focus on our CBK-related work? What is the governance supporting the artifact we are generating? How might we make that artifact equitable, in its creation, deployment, and future management?
5. LIS professionals, must ask, how do we apply open science frameworks to this work, such that transparency and visibility into how the artifact was created and is deployed is fundamental to the effort?
6. LIS professionals must also ask themselves; how can they learn about AI, ML and other advanced technologies and their implications and potential to enhance health equity? How might they apply their skills in metadata schema and ontology development, data management and curation, semantics and the relationships between knowledge artifacts, repositories and iteration controls, and knowledge dissemination, to be part of the future of healthcare and biomedical research that will largely be informed by AI and ML technologies?

## CONCLUSIONS

Readers wishing to learn more and engage in the MCBK community are encouraged to contact the authors or visit the University of Michigan's Learning health System's MCBK website at: https://mobilizecbk.med.umich.edu/. By actively participating in the Mobilizing Computed Biomedical Knowledge community, librarians can contribute their expertise, promote information literacy, and facilitate access to resources, thereby fostering a more inclusive and collaborative research environment.
